# In Vitro Assessment of Essential Oils as Sustainable Antifungal Agents Against *Sclerotinia sclerotiorum* Causing Lettuce Drop

**DOI:** 10.3390/molecules31040682

**Published:** 2026-02-16

**Authors:** Mehdiye Tunç, Simone Piancatelli, Sarah Mojela Makau, Annamaria Lucrezia D’Ortenzio, Marwa Moumni, Sara Simonetti, Laura Papi, Eleonora Grassi, Francesco Bucci, Gianfranco Romanazzi

**Affiliations:** 1 Department of Agricultural, Food and Environmental Sciences, Marche Polytechnic University, Via Brecce Bianche, 60131 Ancona, Italy; m.tunc@pm.univpm.it (M.T.); s.piancatelli@staff.univpm.it (S.P.); sm.makau@tuks.co.za (S.M.M.); a.l.dortenzio@staff.univpm.it (A.L.D.); m.moumni@staff.univpm.it (M.M.); 2 Verde Naturale di Simonetti Sara, Via Ripa 3, 60013 Corinaldo, Italy; info@verdenaturale.it; 3 Undicesimaora Social Cooperative Society, 60019 Senigallia, Italy; laura.papi.7@gmail.com (L.P.); francesco.bucci@gmail.com (F.B.); 4 Department of Biomolecular Sciences, Università di Urbino Carlo Bo, Via Ca’ le Suore 2-4, 61029 Urbino, Italy; eleonora.grassi@uniurb.it

**Keywords:** mycelia growth, *Rosmarinus officinalis*, phytotoxicity, soilborne disease, plant pathogen

## Abstract

*Sclerotinia sclerotiorum* is a soilborne fungal pathogen, and it is a major threat to lettuce production, causing lettuce drop. This study evaluated the antifungal effectiveness of five essential oils (EOs) (*Rosmarinus officinalis*, *R. officinalis* var. *verbenone*, *Lavandula hybrida*, *Origanum majorana*, and *Thymus vulgaris*) at 0.1%, 1%, and 10%, along with their phytotoxic effect in the field on three different crops (lettuce, tomato, and chard) following foliar application. *T. vulgaris* EO completely inhibited *S. sclerotiorum* mycelial growth at all tested concentrations. *R. officinalis*, *L. hybrida*, and *O. majorana* also showed full inhibition at 1% and 10%, while *R. officinalis* var. *verbenone* achieved 80–100% inhibition. *R. officinalis* had the least phytotoxic effects, with only a minimal effect on chard at 1%. *R. officinalis* var. *verbenone* caused low/moderate phytotoxicity in lettuce and chard but had no toxic effect on tomato. *L. hybrida* and *O. majorana* had moderate to low effects, while *T. vulgaris* was the most phytotoxic, significantly affecting lettuce and tomato at 1%. Further field trials are needed to define EO application protocols toward sustainable lettuce drop management without risks of phytotoxicity.

## 1. Introduction

Lettuce (*Lactuca sativa*) is an important salad crop that is produced all over the world [1]. According to FAO estimates, global lettuce production is led by China, with approximately 14.98 million tonnes, followed by India at 1.16 million tonnes. In Europe, the key producers are Spain and Italy, yielding 969,190 tonnes and 638,180 tonnes, respectively [2].

Lettuce crops suffer yield losses due to *Sclerotinia* spp., typically ranging from 10% to 45%, with severe cases reaching up to 70% under favorable conditions for the pathogen [3,4,5]. *Sclerotinia minor* and *Sclerotinia sclerotiorum* are the main species causing lettuce drop [6]. *S. minor*, a soilborne pathogen, infects lettuce roots and leaves via mycelia and can also contaminate seeds under certain conditions. *S. sclerotiorum* primarily infects lettuce plants through ascospores but can also contaminate seeds in favorable environments [7]. *S. sclerotiorum* infections typically begin near the stem, leading to root rot and a watery appearance of the crop [8,9]. *S. minor* infection is characterized by watery decay, browning, and plant collapse due to crown tissue damage with white mycelial mats forming small sclerotia on underground tissues. Infection by *S. minor* starts with sclerotia germination, unlike the airborne spread typical of *S. sclerotiorum* [10]. They can be distinguished by sclerotia size, with *S. minor* producing smaller (1–2 mm) and *S. sclerotiorum* larger (3–10 mm) sclerotia [11,12]. While *S. sclerotiorum* has a wide host range, *S. minor* is more host specific [13]. Secondary spread occurs when diseased tissues contact healthy tissues on adjacent plants. These fungal pathogens threaten lettuce production, causing significant losses, characteristic decay and wilting symptoms, with variations in their life cycles [14,15]. The management of *Sclerotinia* spp. has traditionally relied on the use of synthetic fungicides, with azoxystrobin, fludioxonil, and tebuconazole being among the most used agents [16,17]. These fungicides have proven effective in controlling *Sclerotinia* spp. across various plant species, but widespread use has raised significant environmental concerns about sustainability challenges [18]. In alignment with the European Green Deal and Farm to Fork Strategy, reducing dependence on synthetic pesticides has become mandatory for future agriculture, and many widely used fungicides are facing heavy limitations, leaving growers with fewer tools against crop diseases [19,20]. Consequently, it is important to search for alternative, environmentally friendly strategies for managing *Sclerotinia* spp. and focus on moving toward more sustainable farming practices.

These alternatives encompass biological control agents [21], basic substances [22], and the evaluation of plant extracts, including their nanoforms [23]. Among these, essential oils (EOs) are mixtures of natural organic compounds with inherent antifungal and antibacterial properties. They also stimulate host defenses, enhancing disease control on plants [24]. *Rosmarinus officinalis* EO demonstrated significant antifungal properties against pathogenic fungi, including *Sclerotinia* species. *R. officinalis* EO was evaluated in vitro and field testing, revealing its capacity to effectively inhibit the activity of *Sclerotinia* spp. on various plants [25,26]. *Thymus vulgaris* EO is known for its antifungal properties due to its composition of several bioactive compounds [27]. Previous studies have reported that EOs of *Thymus* spp. and *Lavandula* spp. exhibited dose-dependent antifungal activity against *Sclerotinia* spp. isolates obtained from different sources [28]. EOs are generally regarded as safe (GRAS) compounds for the environment due to their natural origins, rapid degradation, and low toxicity. The phytotoxicity of EOs is a complex phenomenon influenced by their chemical composition and application conditions [29]. Lettuce is the primary test organism due to its rapid germination and documented sensitivity to phytotoxic compounds, making it a standard bioassay species for preliminary toxicity screening [30]. Tomato (*Solanum lycopersicum*), a globally significant crop with significant postharvest losses caused by fungal pathogens, requires that any antifungal treatment be non-phytotoxic to preserve fruit quality and marketability [31,32]. Swiss chard (*Beta vulgaris* subsp. *cicla*), characterized by its delicate leaf structure and pronounced sensitivity to environmental and chemical stressors, serves as an effective bioindicator for detecting subtle phytotoxic effects. Including these contrasting species allowed for a broader and more realistic assessment of EO safety across horticultural systems. Furthermore, within the European Union regulatory framework, the “basic substances” concept under Regulation (EC) No 1107/2009 offers a pathway for authorizing low-risk, naturally derived compounds [33]. Several EOs and plant-based extracts, such as onion oil, grape seed extract, *Equisetum arvense* L., and sweet orange EO, are already approved under this category [34]. Demonstrating both effectiveness and safety could enable the use of EOs, supporting their integration into sustainable plant protection programs.

The objectives of this study were (i) to investigate and compare the in vitro antifungal activity of five different EOs with three different concentrations to control *Sclerotinia* spp., and (ii) to assess the phytotoxic effects of the five EOs on lettuce, tomato and chard under field conditions, using concentrations that maintained antifungal activity in vitro, in order to minimize potential phytotoxicity.

## 2. Results

### 2.1. Sclerotinia *spp.* Isolates from Lettuce Sample

**Figure 1:**
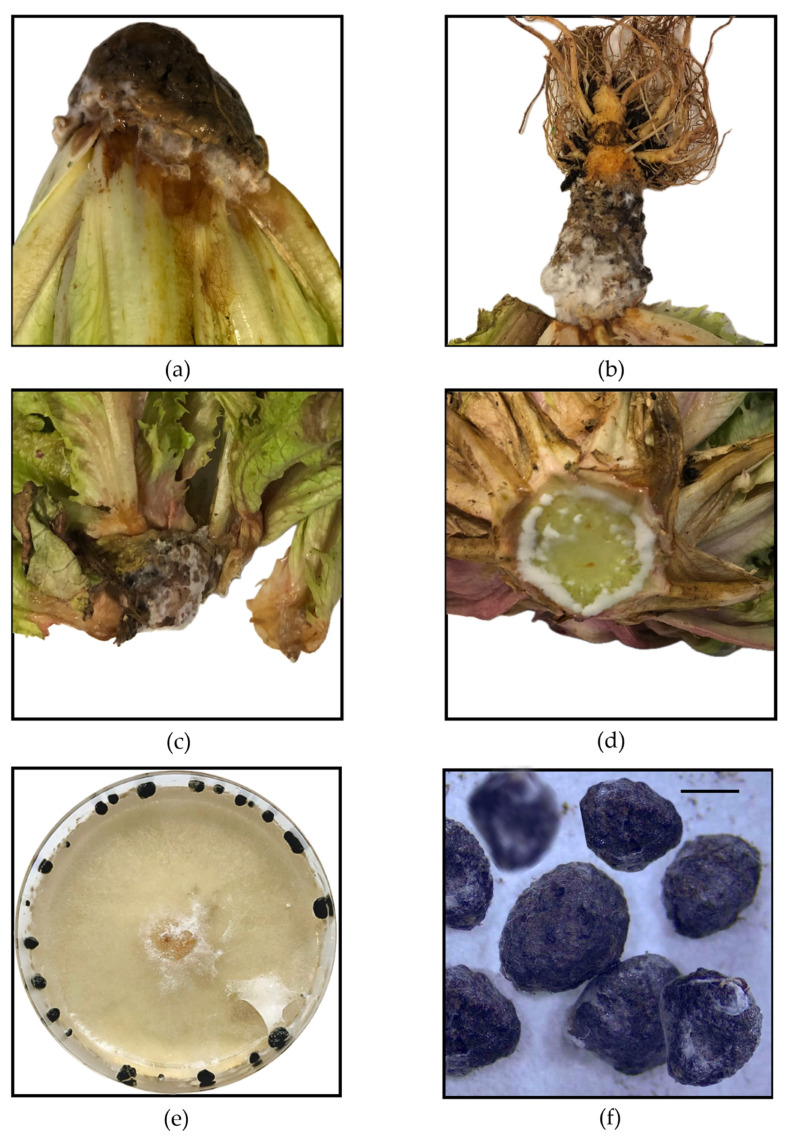
(**a**) Lettuce sample exhibiting characteristic symptoms of *Sclerotinia sclerotiorum* infection, including white cottony mycelium and water-soaked lesions near the base. (**b**) Advanced infection with extensive mycelial growth and black sclerotia formation on the roots. (**c**) Necrotic and decayed lettuce leaves with visible sclerotia embedded in the infected tissue. (**d**) Cross-section of an infected lettuce stem showing internal tissue colonization and soft rot. (**e**) *Sclerotinia sclerotiorum* isolate grown on PDA medium for seven days, displaying characteristic mycelial growth and sclerotia formation along the Petri dish edges. (**f**) Black sclerotia observed under a stereomicroscope. Scale bar: 100 µm.

Among seven suspected lettuce samples, four were confirmed to be infected with *S. sclerotiorum* through morphological identification of the isolated causal organism. Infected lettuce showed severe root and basal stem rot, with water-soaked, soft and mushy tissues. The outer surface appears discolored and becomes brown and dark as the rot progresses. A dense, white, cottony mycelium covers the affected areas and spreads rapidly. Inside the hollow stem, the tissue is completely degraded, leaving a soft, collapsed structure. Small, initially transparent sclerotia begin to form within the decayed tissue, later maturing into hard, black structures. Lettuce isolates of *Sclerotinia* spp. exhibited rapid growth on PDA medium, producing prolific, white, cottony mycelia that completely covered the Petri dishes within two to three days at 21 ± 2 °C. Mycelia initially appeared soft and fluffy, but became more compact and regular as growth progressed. Sclerotia began to form, first appearing as small, transparent structures and gradually darkening into black, hard, irregularly shaped bodies (Figure 1). Mature sclerotia exhibited a hard outer surface with a consistent size range. Morphological analysis of 51 sclerotia, including measurements under the microscope, confirmed the identification of the species. Sclerotium sizes were recorded among the four lettuce isolates and varied slightly between isolates, with measurements presented in Table 1. The treatments were inoculated using these four isolates, referred to as Sc1, Sc2, Sc3, and Sc4.

### 2.2. In Vitro Inhibition of Fungal Growth by the Five Essential Oils

**Figure 2:**
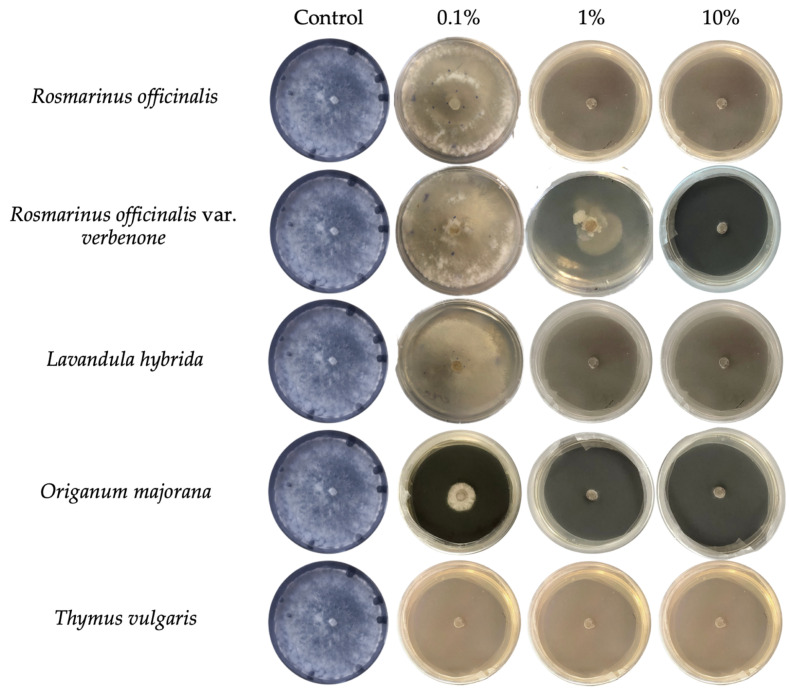
Representative experiment showing the mycelial growth of *Sclerotinia sclerotiorum* isolate 1 after treatment with five essential oils (*Rosmarinus officinalis*, *R. officinalis* var. *verbenone*, *Lavandula hybrida*, *Origanum majorana*, and *Thymus vulgaris*) at 0.1%, 1%, and 10%, compared to the control (PDA amended with Tween 20). Pictures were taken after seven days of plate incubation at 22 ± 2 °C.

The antifungal activity of five EOs was evaluated against four *S. sclerotiorum* isolates (Sc1–Sc4) at three concentrations (0.1%, 1%, and 10%), as can be seen in Figure 2 and Table 2. Inhibition percentages were calculated relative to the control (8.3 cm mycelial growth). The findings demonstrate varied degrees of antifungal activity among the EOs, with a general trend of higher concentrations correlating with higher inhibition of fungal growth in a dose-dependent manner. *T. vulgaris* EO was the most effective, achieving complete inhibition (100%) against all isolates at all concentrations tested, including 0.1%. At 1% and 10% concentrations, *R. officinalis* EO achieved complete inhibition (100%) across all isolates. *R. officinalis* EO has shown limited antifungal activity at 0.1%, with an inhibition percentage of 29.3% and 13.2% against Sc2 and Sc3, respectively, while low inhibition ranging from 0 to 1.0% was observed against Sc1 and Sc4. At 10%, *R. officinalis* var. *verbenone* EO achieved 100% inhibition across all isolates. At 1%, inhibition ranged from 57.0% for Sc1 to 68.8% for Sc4, with complete inhibition (100%) recorded for Sc2 and Sc3. At 0.1% concentration, partial inhibition was observed at 26.30% and 27.7% for isolates Sc2 and Sc3, respectively. In contrast, isolates Sc1 and Sc4 exhibited minimal inhibition, with values ranging from 0% to 1%. At 1% and 10%, *L. hybrida* EOs completely inhibited (100%) fungal growth across all isolates. The same EO demonstrated moderate antifungal activity at 0.1%, achieving inhibition values of 56.2% and 56.9% against Sc2 and Sc3, respectively, 1.0% against Sc4, and low inhibition against Sc1. At both 1% and 10%, *O. majorana* EO achieved complete inhibition (100%) across all isolates. *O. majorana* EO exhibited notable antifungal activity at 0.1%, achieving inhibition values of 56.4%, 62.6%, 90.9%, and 100% against Sc1, Sc4, Sc3, and Sc2, respectively.

### 2.3. Fungicidal and Fungistatic Effects of Essential Oils Against Sclerotinia sclerotiorum

The fungicidal and fungistatic activities of five EOs (*Rosmarinus officinalis*, *R. officinalis* var. *verbenone*, *Lavandula hybrida*, *Origanum majorana*, and *Thymus vulgaris*) were evaluated on four *S. sclerotiorum* isolates (Sc1–Sc4), at concentrations of 0.1%, 1%, and 10% (*v*/*v*). The results are summarized in Table 3. *R. officinalis* EO exhibited a concentration-dependent pattern. At 1%, all isolates that showed complete growth inhibition were able to regrow after re-inoculation, indicating a fungistatic effect. At 10%, a fungicidal effect was observed against isolates Sc3 and Sc4. *R. officinalis* var. *verbenone* EO mainly displayed fungistatic activity. At 10%, fungicidal action was recorded only for Sc3 and Sc4, while the remaining isolates showed regrowth, confirming their predominantly fungistatic nature. *L. hybrida* EO showed a mixed response depending on both concentration and isolate. At 1%, isolates Sc1 and Sc2 exhibited fungistatic behavior, while Sc3 and Sc4 were completely inhibited, showing a fungicidal effect. At 10%, all isolates exhibited fungicidal activity. *O. majorana* EO exhibited both fungistatic and fungicidal effects depending on the concentration and isolate. At 0.1%, it was mostly fungistatic, while at 1% and 10%, fungicidal activity was observed on several isolates. *T. vulgaris* EO was the most active among the tested oils, showing fungicidal effects against all isolates at all concentrations tested.

### 2.4. Phytotoxicity Evaluation of the Five Essential Oils in Lettuce, Tomato, and Chard

**Figure 3:**
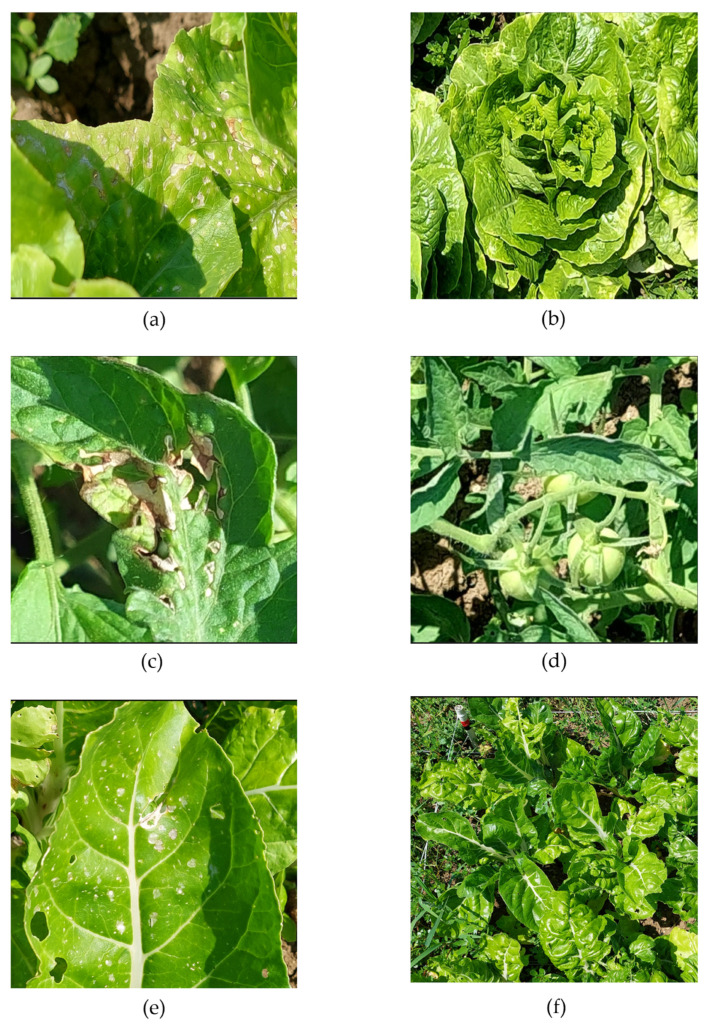
Representative phytotoxic symptoms caused by *Thymus vulgaris* EO (1%), the most phytotoxic treatment, showing yellow spots, leaf burn, and necrosis on (**a**) lettuce compared with (**b**) control, (**c**) tomato compared with (**d**) control, and (**e**) chard compared with (**f**) control. Additional pictures for the other essential oils are presented in Figure S2.

Visible phytotoxic responses were observed on lettuce, tomato and chard treated with five EOs (*Rosmarinus officinalis*, *R. officinalis* var. *verbenone*, *Lavandula hybrida*, *Origanum majorana*, and *Thymus vulgaris*) at concentrations of 0.1 and 1% (*v*/*v*). Photos of lettuce plants treated with different EOs are reported in Figure 3 and Figure S1 and Table 4 to summarize the results of the phytotoxicity evaluation for all the crops.

#### 2.4.1. Lettuce

In lettuce, plants treated with *R. officinalis* EO showed no visible phytotoxicity at either concentration (0.1% and 1%); leaves remained similar to those of the untreated control plants (Figure S1a,b). Plants treated with *R. officinalis* var. *verbenone* EO caused low phytotoxicity at both concentrations, affecting up to 20% of plants with small, scattered yellow spots on the leaf surface (Figure S1c,d). Using *L. hybrida* EO, clear differences were observed between concentrations. At 0.1%, medium phytotoxicity symptoms were visible, with approximately 20–50% of plants exhibiting yellow spots and slight leaf burn, at 1%, the phytotoxicity level was lower, affecting up to 20% of plants (Figure S1e,f). No visible symptoms were detected in plants treated with *O. majorana* EO at all concentrations, and their general appearance was like the untreated controls (Figure S1g,h). Plants treated with *T. vulgaris* EO showed the highest level of phytotoxicity among all treatments. At 1%, over 50% of the plants were severely affected, exhibiting widespread yellow spots, leaf burn (Figure S1i,j), while no symptoms were visible at 0.1%. All treatments were compared with the untreated control plants (Figure S1k,l).

#### 2.4.2. Tomato

In tomato plants, no visible phytotoxicity was detected in any of the treatments with *R. officinalis*, *R. officinalis* var. *verbenone*, or *L. hybrida* EOs at both concentrations (0.1% and 1%). Similarly, *O. majorana* EO did not cause visible phytotoxic symptoms at 0.1%, and only a few plants showed low phytotoxicity at 1%, with yellow spots observed on some leaves during the second assessment. In contrast, plants treated with *T. vulgaris* EO at 1% exhibited high phytotoxicity, affecting more than 50% of the plants, which displayed widespread yellow spots, leaf burn, and necrosis. At 0.1%, no visible phytotoxicity symptoms were observed, and treated plants remained comparable to the controls. *T. vulgaris* EO was the only treatment that induced a severe phytotoxic response in tomato, while the other oils were well tolerated by the plants.

#### 2.4.3. Chard

In chard plants, phytotoxic responses varied depending on the EO and concentration applied. Plants treated with *R. officinalis* EO showed no visible phytotoxicity at 0.1%, whereas low phytotoxicity was observed at 1% during the second assessment, with up to 20% of plants displaying scattered yellow spots on the leaf surface. Treatment with *R. officinalis* var. *verbenone* EO caused medium phytotoxicity at both concentrations, affecting 20–50% of plants, which showed yellow spots and slight leaf burns. In plants treated with *L. hybrida* EO, the phytotoxic response increased with concentration, with low phytotoxicity observed at 0.1%, and medium symptoms developed at 1%, with up to 50% of plants showing partially burnt leaves. *O. majorana* and *T. vulgaris* EOs both induced low phytotoxicity at 0.1% and 1%, with 1–20% of plants showing small yellow spots and minor leaf burn, but no severe damage was detected. *R. officinalis* var. *verbenone* and *L. hybrida* EOs produced the most evident phytotoxic effects in chard, while *O. majorana* and *T. vulgaris* EOs were the least harmful treatments.

## 3. Discussion

*S. sclerotiorum* is a highly destructive fungal pathogen, causing significant losses in lettuce production worldwide. Considering the limitations of conventional control methods, the research of alternative strategies is crucial for sustainable disease management. Morphological identification provides an initial approach for characterizing *S. sclerotiorum*, confirming its colony morphology, hyphal structure, and sclerotia formation [35,36,37]. In our study, microscopic analysis confirmed the presence of septate hyphae with characteristic branching patterns [38]. Sclerotia size measurements across all four isolates showed uniformity, ranging from 2.5 to 2.8 mm, which aligns with previous studies. This consistency supports the reliability of morphological identification methods for diagnosing *S. sclerotiorum* [39,40,41].

Our in vitro findings demonstrate the effectiveness of EOs tested and provide a new insight beyond simple inhibition. A key result was that *T. vulgaris* EO completely inhibited all *S. sclerotiorum* isolates at all tested concentrations (0.1%, 1% and 10%). This broad-spectrum activity is consistent with its fungicidal properties, which can be explained by the high content of phenolic compounds such as thymol and carvacrol, which are known to disrupt cell membranes and metabolic pathways [42,43]. *O. majorana* EO was the second most effective treatment, exhibiting dose-dependent activity that varied between isolates. Sc2 was inhibited at 0.1% while Sc3 and Sc4 required higher concentrations for fungicidal effects. This observation aligns with previous findings, which reported dose-dependent antifungal activity of *O. majorana* EO against various fungal pathogens [44]. The activity of *R. officinalis* and *L. hybrida* EOs was strongly concentration-dependent, with consistent fungicidal activity achieved only at 1% and 10%. The requirement for a higher concentration of *R. officinalis* var. *verbenone* EO in our study, compared to reports of complete inhibition at 0.1% for other chemotypes, suggests that the antifungal activity is linked to the specific chemical profile of the EO. This chemotypic variation is a key factor explaining variation between studies and emphasizes the need for standardized chemical characterization in bioactivity research [45,46]. The chemical composition of each EO plays an important role in determining its mechanism of antifungal activity. As reported in Table 5, the EOs of *R. officinalis* and *L. hybrida* are particularly rich in key compounds such as α-pinene, 1,8-cineole, camphene, and limonene. According to previous studies, oxygenated monoterpenes, including α-pinene, linalool, and 1,8-cineole, which are dominant constituents in *L. hybrida* and *R. officinalis* EOs, have demonstrated antifungal activity against major plant pathogens like *Alternaria alternata* and *Fusarium oxysporum* [47,48]. Phenolic monoterpenes such as thymol and carvacrol, which are abundant in *T. vulgaris* EO, are widely recognized as highly potent antifungal agents. Their effectiveness against destructive pathogens such as *Botrytis cinerea* and *S. sclerotiorum* has been extensively reported [49,50]. These compounds express their antifungal action mainly through the disruption of fungal cell membranes, leading to leakage of intracellular contents and ultimately inhibiting fungal growth [49,50].

The core innovation of our study lies in the direct linkage of antifungal effectiveness with a simultaneous, multi-crop phytotoxicity assessment. This integrated approach is important for identifying EOs with a viable safety profile. Studies have demonstrated that the phytotoxic effects of EOs vary depending on plant species, developmental stages, and concentrations [51]. The most potent fungicide, *T. vulgaris* EO, has the highest phytotoxic risk to lettuce and tomato at 1% concentration. *R. officinalis* EO demonstrated the most promising balance, combining significant antifungal activity with minimal phytotoxicity, causing only slight symptoms on chard at 1%, a finding supported by its relative safety on other crops [52,53]. *O. majorana* and *L. hybrida* induced low to moderate effects that varied by crop species. A particularly significant and novel finding was the high sensitivity of chard to most EOs, a previously unreported phenomenon that provides crucial new information for growers of this crop [54,55]. This underlines that phytotoxicity is not a universal trait but is highly specific to both the EO and the crop species, necessitating individual evaluation.

Our research goes beyond simple in vitro screening, providing an integrated risk-benefit analysis. We identified *T. vulgaris* as the most potent antifungal EO, but its phytotoxic risk limits its practical use. In contrast, *R. officinalis* and *O. majorana* EOs represent the most promising candidates, offering effective pathogen control with an acceptable safety profile for tomato and lettuce. The practical importance of these results is significantly amplified within the regulatory framework of the European Union for “basic substances” (Regulation (EC) No 1107/2009). This classification is designed for safe, naturally occurring substances mainly used for purposes other than plant protection (for example, as foodstuffs or medicinal products). EOs are good candidates for this pathway since they are considered generally recognized as safe (GRAS) compounds. The approval of EOs and other plant extracts, such as onion oil, garlic extract, and mustard seed powder, as basic substances, provides a clear background. Obtaining basic substance status for the EOs identified in this study has profound implications. Firstly, it offers a faster and more cost-effective route to commercialization compared to conventional synthetic pesticides. Secondly, basic substances count as “0” in the calculation of the Harmonized Risk Indicator 1 (HRI-1), a key parameter for the EU directive on sustainable use of pesticides. This provides a strong incentive for farmers to adopt these low-risk alternatives and contributes to achieving policy goals for reducing the use of chemical pesticides. Therefore, our work lays the essential groundwork for future applied research. The promising effectiveness and safety profile of EOs justifies immediate field validation to confirm their performance under real conditions. This research pipeline, from in vitro effectiveness and phytotoxicity to field studies, is fundamental for creating the scientific dossier required to support future applications of these EOs as potential basic substances, thereby expanding the range of sustainable and accessible options for modern agriculture.

## 4. Materials and Methods

### 4.1. Isolation of Lettuce

Seven lettuce (*Lactuca sativa* var. *longifolia*) samples, suspected of *Sclerotinia* spp. infection, were obtained from the Marche region, Italy. Five lettuce samples were from greenhouse cultivation, and two were from field cultivation. All samples were examined under a stereomicroscope (Leica M125, Leica Microsystems CMS, Wetzlar, Germany) to verify the presence of *Sclerotinia* spp. by assessing the presence of sclerotia or mycelia. Each affected plant was sliced into small fragments (less than 5 mm) using flame-sterilized scalpels. These fragments were immersed in 1% sodium hypochlorite for 1 min, rinsed three times with sterile distilled water, and dried on sterile blotter paper in a laminar flow hood. The fragments were then placed on potato dextrose agar (PDA, 42 g/L; Liofilchem Srl, Roseto degli Abruzzi, Italy) and incubated at 22 ± 2 °C for seven days. The plates were checked daily, and colonies grown were transferred to fresh PDA plates to obtain pure *Sclerotinia* spp. cultures. Morphological identification was performed by growth type, sclerotia characteristics, and the colors and shapes of the colonies. Fungal structures were measured using LAS V3.8 software (Leica DFC 295) from 51 units of each structure for each fungal isolate. Species identification was conducted using the taxonomic keys provided [36,37].

### 4.2. In Vitro Antifungal Activity on Mycelial Growth

The antifungal activity of five EOs (*R. officinalis*, *R. officinalis* var. *verbenone*, *L. hybrida*, *O. majorana* and *T. vulgaris*) was assessed against four *S. sclerotiorum* isolates derived from various lettuce samples. EOs were purchased from Verde Naturale Company (Corinaldo, Ancona, Italy). According to the manufacturer, the EOs were 100% pure and obtained by steam distillation (Table 5). The EOs were kept in sealed amber vials and stored at 4 °C until used in all experiments. The EOs were assessed against four *S. sclerotiorum* isolates derived from various lettuce samples. To prepare homogeneous emulsions, EOs were dissolved in sterile distilled water containing 0.1% (*v*/*v*) Tween 20 (Sigma Aldrich, Steinheim, Germany). The emulsions were then incorporated into potato dextrose agar (PDA) medium, cooled to 40 °C, to achieve final EO concentrations of 0.1%, 1%, and 10%. Petri dishes (90 mm in diameter) were filled with 15 mL of PDA amended with EO emulsions, while PDA containing only 0.1% (*v*/*v*) Tween 20 served as the control. Each Petri dish was inoculated with a 6 mm plug of *S. sclerotiorum*, and five replicates were prepared for each EO concentration. Plates were sealed with parafilm and incubated at 22 ± 2 °C for seven days. Fungal colony diameters (measured perpendicularly) were recorded daily until mycelial growth reached the edge of the control plates. Mycelial growth inhibition was calculated using Equation (1):

(1) $$Mycelial\;Growth\;Inhibition\left(\%\right)=\left(\frac{\left(dMc-dMt\right)}{dMc}\right)\times 100,$$

where “dMc” is the mean diameter of the control colonies and “dMt” is the mean diameter of the treated colonies.

To differentiate between fungicidal and fungistatic effects, the methodology outlined by Moumni et al. [56] was followed. Mycelial plugs showing complete growth inhibition on EO-amended media were transferred onto fresh PDA plates without EOs and incubated at 22 ± 2 °C for seven days. The absence of fungal regrowth was considered indicative of a fungicidal effect, whereas resumed mycelial growth was interpreted as fungistatic activity.

### 4.3. Phytotoxicity Assessment of the Five Essential Oils in Lettuce, Tomato and Chard

Phytotoxicity of the EOs was evaluated following foliar application in open-field conditions in Senigallia, Italy (Latitude: 43.697778° N; Longitude: 13.242778° E). Lettuce, tomato, and chard plants were transplanted on 1 June 2023, and treatments were carried out 22 days later. EOs were applied at concentrations of 0.1% and 1%, selected based on confirmed effectiveness in in vitro studies, to assess potential side effects. Treatments were applied to one plot per treatment per crop, with each plot containing nine plants. A spraying volume of 1 L per plot was used. Control plots were sprayed with water containing 0.1% Tween 20.

Phytotoxicity assessments were conducted on 2 July 2023 (11 days after application) and 14 July 2023 (22 days after application). Phytotoxicity was assessed visually on individual plants by observing foliar changes such as yellow spotting and leaf burning in comparison with untreated controls. In several cases, these symptoms were associated with reduced plant growth, and in the most severe instances, plant death was observed. The extent of phytotoxic effects was classified using an empirical scale: no symptoms (NS); low (1–20% of plants showing phytotoxicity); moderate (21–50%); and high (>51%).

### 4.4. Statistical Analysis

Data were expressed as mean ± standard deviation (SD) and analyzed using SPSS software (version 21.0; SPSS Inc., Chicago, IL, USA). Analysis of variance (ANOVA) was performed to determine significant differences among treatments, and means were compared using Tukey’s test at a significance level of *p* ≤ 0.05.

## 5. Conclusions

This research highlighted the potential of five EOs, *R. officinalis*, *R. officinalis* var. *verbenone*, *L. hybrida*, *O. majorana*, and. *T. vulgaris*, in inhibiting the mycelial growth of *S. sclerotiorum. T. vulgaris* EO demonstrated complete inhibition of mycelial growth across all tested isolates and exhibited consistent fungicidal activity at all tested concentrations, marking it as a highly effective candidate for disease management. At a low concentration (0.1%), *T. vulgaris* EO showed no phytotoxic effects on lettuce, reinforcing its potential for safe application. Its effectiveness at low concentration is particularly significant given the high cost of EOs and the risks of phytotoxicity on fresh produce, making it a promising and economically viable alternative to synthetic fungicides.

Future research should build upon these findings by assessing the performance of these EOs beyond controlled conditions, particularly in greenhouse and field trials, to further validate their effectiveness and practical applicability. As the demand for sustainable agricultural solutions grows, EOs offer a compelling, eco-friendly alternative for controlling *S. sclerotiorum* infections.

## Figures and Tables

**Table 1:** Average sclerotium diameter in micrometers (µm) and standard deviation among lettuce isolates.

**Code of Isolates**	**Source of Isolate**	**Average Diameter (µm)**
Sc1	Root of plant 1	272.2 ± 32.3
Sc2	Root of plant 2	251.6 ± 40.2
Sc3	Root of plant 3	285.5 ± 40.2
Sc4	Root of plant 4	284.6 ± 14.3

**Table 2:** Mycelial radial growth (cm) of four *Sclerotinia sclerotiorum* isolates treated with five essential oils. Measurements were recorded after 7 days of inoculation at 22 ± 2 °C, when the colonies of the control reached the edge of the Petri dishes.

Treatments (Concentration%)	Mycelial Growth (cm)			
	Sc1	Sc2	Sc3	Sc4
*Rosmarinus officinalis* 0.1%	8.3 ± 0.0 a *	5.8 ± 0.6 b	7.2 ± 0.8 ab	5.7 ± 1.0 b
*Rosmarinus officinalis* 1%	0.0 c	0.0 d	0.0 d	0.0 d
*Rosmarinus officinalis* 10%	0.0 c	0.0 d	0.0 d	0.0 d
*Rosmarinus officinalis* var. *verbenone* 0.1%	8.3 ± 0.0 a	6.1 ± 0.5 b	6.0 ± 0.4 b	7.0 ± 0.8 a
*Rosmarinus officinalis* var. *verbenone* 1%	3.5 ± 1.3 b	0.0 d	1.2 ± 0.7 d	0.0 d
*Rosmarinus officinalis* var. *verbenone* 10%	0.0 c	0.0 d	0.00 d	0.0 d
*Lavandula hybrida* 0.1%	8.3 ± 0.0 a	3.6 ± 1.4 c	3.7 ± 1.1 c	8.2 ± 0.1 a
*Lavandula hybrida* 1%	0.0 c	0.0 d	0.0 d	0.0 d
*Lavandula hybrida* 10%	0.0 c	0.0 d	0.0 d	0.0 d
*Origanum majorana* 0.1%	3.6 ± 1.0 b	0.0 d	0.5 ± 0.8 d	2.1 ± 0.3 c
*Origanum majorana* 1%	0.0 c	0.0 d	0.0 d	0.0 d
*Origanum majorana* 10%	0.0 c	0.0 d	0.0 d	0.0 d
*Thymus vulgaris* 0.1%	0.0 c	0.0 d	0.0 d	0.0 d
*Thymus vulgaris* 1%	0.0 c	0.0 d	0.0 d	0.0 d
*Thymus vulgaris* 10%	0.0 c	0.0 d	0.0 d	0.0 d
Control	8.3 ± 0.0 a	8.3 ± 0.0 a	8.3 ± 0.0 a	8.3 ± 0.0 a

* Data are reported as means ± SD. Values with different letters in the same column are significantly different according to one-way ANOVA analysis and Tukey’s test (*p* ≤ 0.05).

**Table 3:** Fungicidal and fungistatic activities of five essential oils (*Rosmarinus officinalis*, *R. officinalis* var. *verbenone*, *Lavandula hybrida*, *Origanum majorana*, and *Thymus vulgaris*) on four *Sclerotinia sclerotiorum* isolates (Sc1–Sc4), at three concentrations (0.1%, 1%, and 10%, *v*/*v*). The effects were determined after seven days of incubation at 22 ± 2 °C.

Treatments (Concentration%)	Sc1	Sc2	Sc3	Sc4
*Rosmarinus officinalis* 0.1%	— *	—	—	—
*Rosmarinus officinalis* 1%	FS **	FS	FS	FS
*Rosmarinus officinalis* 10%	FS	FS	FC ***	FC
*Rosmarinus officinalis* var. *verbenone* 0.1%	—	—	—	—
*Rosmarinus officinalis* var. *verbenone* 1%	FS	FS	FS	FS
*Rosmarinus officinalis* var. *verbenone* 10%	FS	FS	FC	FC
*Lavandula hybrida* 0.1%	—	—	—	—
*Lavandula hybrida* 1%	FS	FS	FC	FC
*Lavandula hybrida* 10%	FC	FC	FC	FC
*Origanum majorana* 0.1%	FS	FS	FS	FS
*Origanum majorana* 1%	FS	FS	FC	FC
*Origanum majorana* 10%	FC	FC	FC	FC
*Thymus vulgaris* 0.1%	FC	FC	FC	FC
*Thymus vulgaris* 1%	FC	FC	FC	FC
*Thymus vulgaris* 10%	FC	FC	FC	FC

*—Not tested ** Fungistatic *** Fungicidal.

**Table 4:** Phytotoxicity of five essential oils (*Rosmarinus officinalis*, *Rosmarinus officinalis* var. *verbenone*, *Lavandula hybrida*, *Origanum majorana*, *Thymus vulgaris*) on lettuce, tomato, and chard plants treated with 0.1% and 1% concentrations (*v*/*v*) and evaluated at 11 and 22 days after application (DAA). Symptoms were assessed individually by plant species, and an overall score was assigned based on the appearance of 9 plants per treatment (NS, no phytotoxic symptoms; +, low phytotoxicity (1–20% of plants showing symptoms); ++, medium phytotoxicity (21–50% of plants showing symptoms); +++, high phytotoxicity (>51% of plants showing symptoms). Changes over time are shown as first/second assessment (e.g., NS/+, if the plant was asymptomatic in the first assessment and started to show symptoms in the second assessment).

Treatments (Concentration%)	Crop		
	Lettuce	Tomato	Chard
*Rosmarinus officinalis* 0.1%	NS	NS	NS
*Rosmarinus officinalis* 1%	NS	NS	NS/+
*Rosmarinus officinalis* var. *verbenone* 0.1%	+	NS	++
*Rosmarinus officinalis* var. *verbenone* 1%	+	NS	++
*Lavandula hybrida* 0.1%	++	NS	+
*Lavandula hybrida* 1%	+	NS	++
*Origanum majorana* 0.1%	NS	NS	+
*Origanum majorana* 1%	NS	NS/+	+
*Thymus vulgaris* 0.1%	NS	NS	+
*Thymus vulgaris* 1%	+++	+++	+

**Table 5:** Essential oils assessed for their in vitro antifungal activity against *Sclerotinia sclerotiorum*. The table reports the plant species of origin, common names, commercial providers, and the main chemical constituents of each essential oil according to the supplier’s specifications.

Plant Species, Concentration	Common Name	Provider	Main Components
*Rosmarinus officinalis*, 100%	Rosemary	Verde Naturale	α-pinene 26.34%, 1,8-cineol 9.57%, camphene 8.35%, limonene 5.82%, verbenone 2.36%, camphor 1.47%, linalool 0.54%
*Rosmarinus* *officinalis* var. *verbenone* 100%	Rosemary verbenone	Verde Naturale	α-pinene 43.45%, limonene 9.73%, verbenone 8.39%, 1,8-cineole 3.91%
*Lavandula hybrida* 100%	Hybrid lavender, lavandin	Verde Naturale	linalool 47.44%, linalyl acetate 29.77%, camphor 4.10%, limonene 3.41%
*Origanum majorana* 100%	Marjoram, Sweet Marjoram	Verde Naturale	terpinen-4-ol 34.22%, γ-terpinene 9.89%, α-terpinene 9.33%, cis-sabinene hydrate 7.04%, linalool 6.30%, and α-terpineol 5.15%
*Thymus vulgaris* 100%	Thyme	Verde Naturale	thymol 28.3%, carvacrol 6.55%, p-Cymen 36.8%, borneol 0.65%, linalool 5.10%

## Data Availability

The data presented in this study are available on request from the corresponding author.

## Supplementary Materials

The following supporting information can be downloaded at: https://www.mdpi.com/article/10.3390/molecules31040682/s1. Figure S1. Effects of essential oils in lettuce culture, (a) *Rosmarinus officinalis* 0.1%, (b) *R. officinalis* 1%, (c) *R. officinalis* var. *verbenone* 0.1%, (d) *R. officinalis* var. *verbenone* 1%, (e) *Lavandula hybrida* 0.1%, (f) *L. hybrida* 1%, (g) *Origanum majorana* 0.1%, (h) *O. majorana* 1%, (i) *Thymus vulgaris* 0.1%, (j) *T. vulgaris* 1%, (k, l) control. Figure S2. Phytotoxicity evaluation of *Rosmarinus officinalis*, *Rosmarinus officinalis* var. *verbenone*, *Lavandula hybrida*, *Origanum majorana*, and *Thymus vulgaris* essential oils on different crops following foliar application. The panel illustrates representative visible morphological responses (including yellow spots, leaf necrosis and growth inhibition) observed on different crops with all the tested essential oils. Each essential oil treatment was evaluated based on span changes according to the different plant and symptom severity.
