# Supplementary material for: In Vitro Assessment of Essential Oils as Sustainable Antifungal Agents Against Sclerotinia sclerotiorum Causing Lettuce Drop
Source: Molecules. 2026 Feb 16;31(4):682. doi: 10.3390/molecules31040682 (PMC12943384; doi:10.3390/molecules31040682)
Supplement: Supplementary file 1 [file molecules-31-00682-s001.zip › molecules-4111798-supplementary.pdf]

Supplementary Material

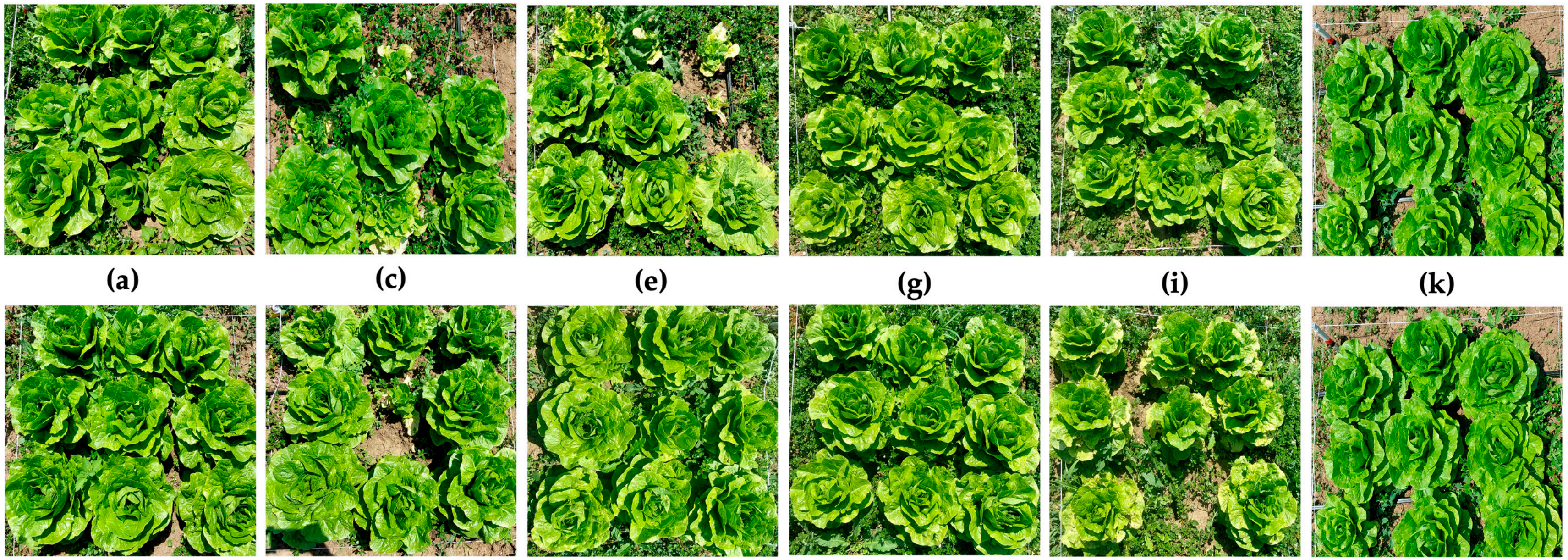

(a) (c) (e) (g) (i) (k)

(b) (d) (f) (h) (j) (l)

Figure S1. Effects of essential oils in lettuce culture, (a) *Rosmarinus officinalis* 0.1%, (b) *R. officinalis* 1%, (c) *R. officinalis* var. *verbenone* 0.1%, (d) *R. officinalis* var. *verbenone* 1%, (e) *Lavandula hybrida* 0.1%, (f) *L. hybrida* 1%, (g) *Origanum majorana* 0.1%, (h) *O. majorana* 1%, (i) *Thymus vulgaris* 0.1%, (j) *T. vulgaris* 1%, (k, l) control

Phytotoxicity symptoms	No symptom	Low (+)	Medium (++)	High(+++)
Rosmarinus officinalis	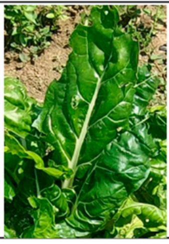	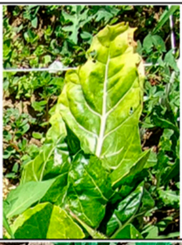		
Rosmarinus officinalis var. verbenone		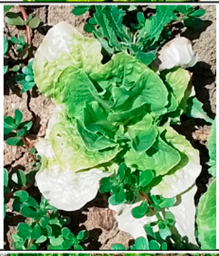	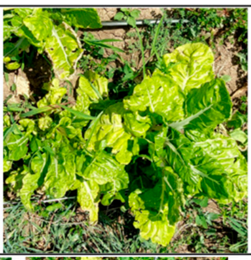	
Lavandula hybrida		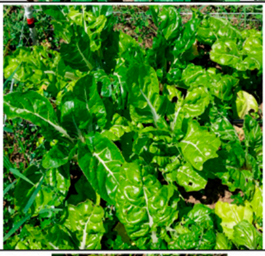	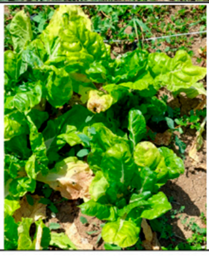	
Origanum majorana		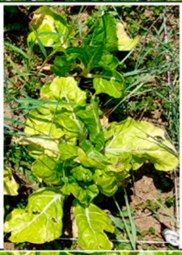		
Thymus vulgaris		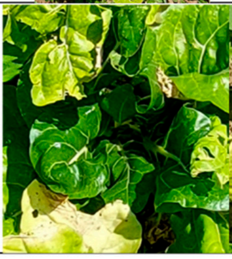	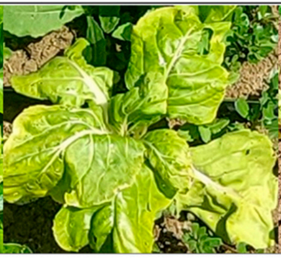	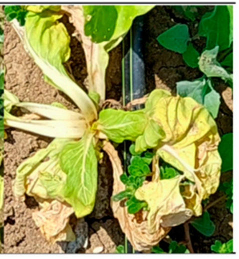

Figure S2. Phytotoxicity evaluation of *Rosmarinus officinalis*, *Rosmarinus officinalis* var. *verbenone*, *Lavandula hybrida*, *Origanum majorana*, and *Thymus vulgaris* essential oils on different crops following foliar application. The panel illustrates representative visible morphological responses (including yellow spots, leaf necrosis and growth inhibition) observed on different crops with all the tested essential oils. Each essential oil treatment

was evaluated based on span changes according to the different plant types and symptom severity.
